# Comprehensive Management of Mandibular Canine Transmigration: A Multidisciplinary Approach

**DOI:** 10.7759/cureus.69738

**Published:** 2024-09-19

**Authors:** Ashish K Singh, Dhruv Ahuja, Puneet Batra, Priya Dogra, Toni Lego

**Affiliations:** 1 Orthodontics and Dentofacial Orthopaedics, Manav Rachna Dental College, Manav Rachna International Institute of Research and Studies, Faridabad, IND

**Keywords:** impaction, mandibular canine, tads, transmigration, uprighting spring

## Abstract

Transmigration of mandibular canine teeth is a rare dental anomaly characterized by the tooth's aberrant migration through the alveolar bone. This deviation from the expected eruption pathway can lead to tooth impaction, root resorption, periodontal problems, and aesthetic concerns. The exact cause of transmigration is not fully understood, but it is believed to be influenced by a combination of genetic, developmental, and environmental factors. Early diagnosis and appropriate treatment are essential to prevent complications and achieve optimal functional and aesthetic outcomes.

This case report presents a 19-year-old female with impacted left mandibular canine teeth. An interdisciplinary approach involving orthodontic therapy and surgical exposure to disimpact the transmigrated canine into the normal occlusal position. The treatment utilized modified orthodontic mechanics, including uprighting springs and temporary anchorage devices (TADs) to treat transmigration. This case also highlights the importance of early diagnosis and timely intervention for transmigrated canine teeth to prevent complications and achieve optimal aesthetic and functional outcomes.

## Introduction

The incidence of impaction in mandibular canines is significantly lower than that of impacted maxillary canines. While mandibular canine impaction ranges from 0.05% to 0.4%, maxillary canine impaction is more prevalent, occurring in 0.9%-2.2% of the cases [1]. Impacted teeth usually stay on the same side of the jaw, but sometimes they can move to a different location. An impacted tooth that crosses over the midline to the other side of the jaw is said to have undergone transmigration [2]. Transmigration is uncommon and only occurs with impacted canines. These teeth can move from one side of the jaw to the other. There is limited information on the transmigration of lateral incisors or premolars [3].

The aetiology and exact mechanism of canine transmigration remain uncertain. However, several factors, including premature deciduous tooth loss, primary canine retention, dental crowding, odontomas, supernumerary teeth, and increased length of canine crowns, are believed to contribute to this phenomenon. Individuals with canine transmigration frequently exhibit additional dental abnormalities, such as missing mandibular lateral incisors or second premolars, enamel developmental issues, fewer teeth overall, or impacted maxillary canines [4].

Canine transmigration is more prevalent on the left side of the mandible than on the right and occurs more frequently in females compared to males in a 1.6:1 ratio. Unilateral transmigration occurs more frequently than bilateral transmigration. Interestingly, bilateral transmigration of impacted mandibular canines can occur even in the presence of adequate eruption space. Transmigration can lead to root resorption, increase sensitivity of neighbouring teeth and potentially causing discomfort or pain for the patient [5].

Mupparapu's classification categorizes transmigrated mandibular canines into five types, with type 1 being the most prevalent. Early detection and evaluation of the degree of transmigrated canine are essential for preventing impaction and mitigating related aesthetic and functional complications [6]. Untreated or improperly managed impacted canines can result in adverse sequelae, including external root resorption of adjacent teeth, particularly the incisors, aesthetic compromise, misalignment of neighbouring teeth, reduced dental arches, increased incidence of cyst formation with impaction, and ultimately, Irremediable damage that may lead to tooth loss [4].

This case study presents a comprehensive approach to managing a transmigrated mandibular canine in a 19-year-old female, involving a collaborative effort between orthodontic and surgical treatment modalities. The treatment plan incorporated modified orthodontic techniques and surgical intervention to successfully address the transmigration.

## Case presentation

**Figure 1:**
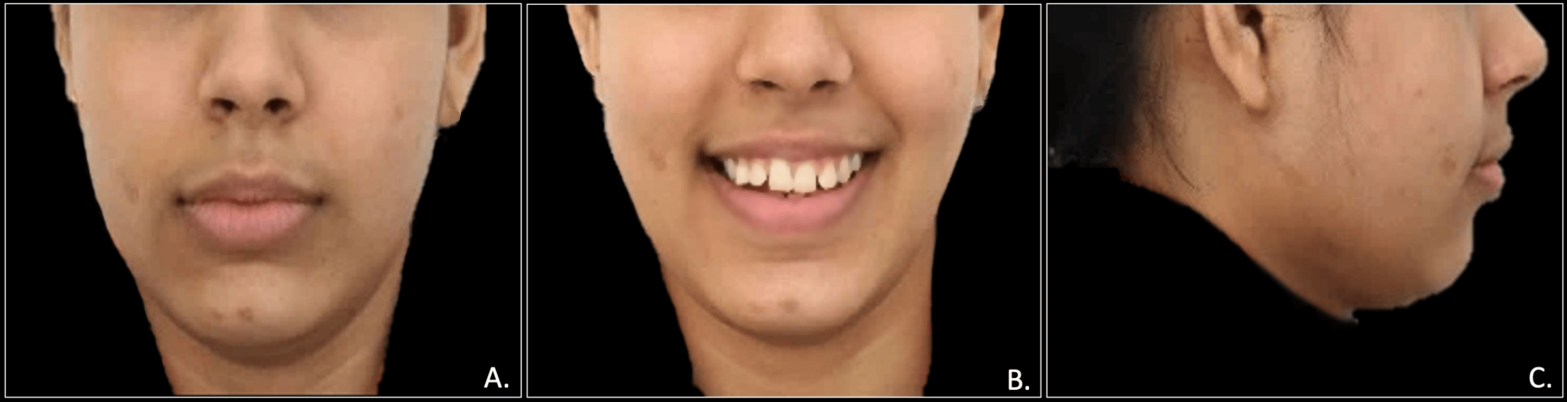
Pre-treatment extraoral photographs: frontal view (A), frontal smiling view (B), and profile view (C)

A 19-year-old female presented to our dental clinic with a concerns of forwardly placed upper front teeth and spacing in both upper and lower teeth. Extraoral examination exhibited a convex facial profile with obtuse nasolabial angle and competent lips (Figure 1).

**Figure 2:**
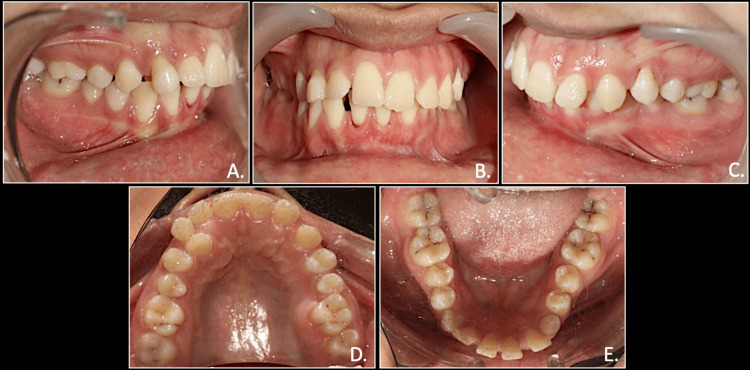
Pre-treatment intraoral photographs: right occlusion (A), anterior occlusion (B), left occlusion (C), maxillary arch (D), mandibular arch (E)

Intraoral examination revealed that the patient had retained deciduous canines in the first and third quadrants with an unerupted canine in the third quadrant and a palatally present canine in the first quadrant. She also had crossbite in relation to the left side with the lower midline shifted toward the right by 1mm. She presented with class II molar on the right and class 1 molar on the left side (Figure 2).

**Figure 3:**
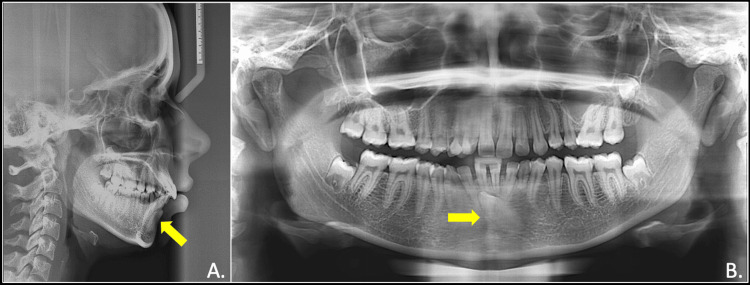
Pre-treatment radiographs showing impacted mandibular canine (yellow arrow): lateral cephalogram (A) and OPG (B) OPG: orthopantomogram

The panoramic radiograph examination showed unfavourable eruption pathway for the mandibular permanent left canines and developing third molars in upper left, lower right, and left quadrants. Cephalometric analysis revealed class I skeletal base and a normodivergent growth pattern with proclined and forwardly placed maxillary and mandibular incisors. The lips were normally placed in relation to the E-line (aesthetic line) (Figure 3).

**Figure 4:**
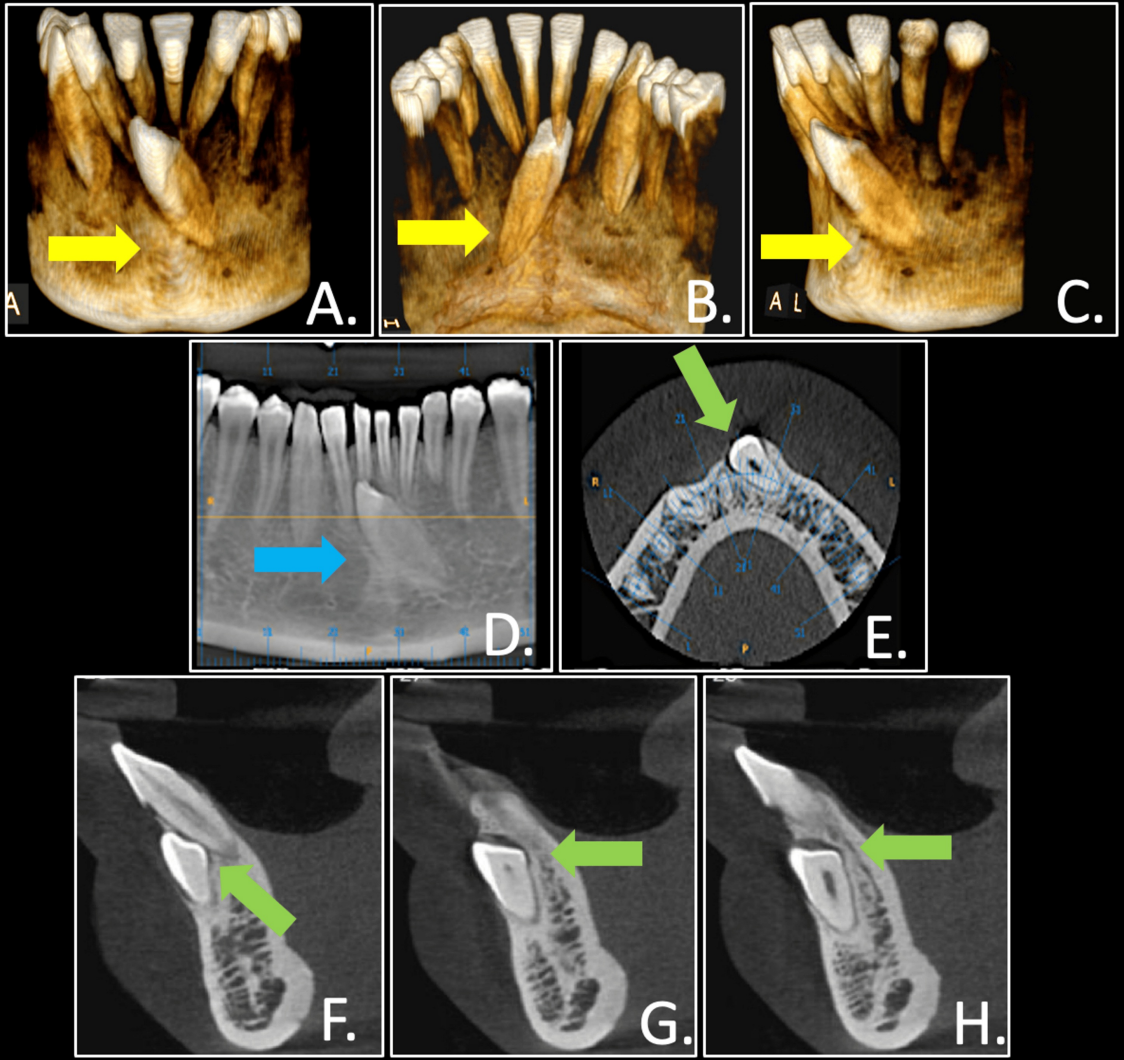
Pre-treatment CBCT scan showing impacted mandibular canine in relation with mandibular incisors and alveolar bone in three dimension: hard tissue reconstruction (A to C) (yellow arrows), reconstructed OPG section focusing on canine impaction (D) (blue arrow), axial and sagittal views (E to H) (green arrows) CBCT: cone beam computed tomography; OPG: orthopantomogram

A cone beam computed tomography (CBCT) scan with 6-FOV (field of view), 0.2 mm voxel size, a 10-second exposure period was obtained. The crown of the impacted canine was positioned on the labial side, below the tips of the mandibular incisors (Figure 4).

Treatment objectives

The main goals of the treatment were to (1) establish a level and aligned dental arch, (2) guide the impacted canine into its correct position within the dental arch, (3) correct the axial inclination of upper and lower incisors, (4) address the scissor bite, and (5) achieve a normal overjet and overbite.

Treatment alternatives

The ideal treatment plan was the extraction of all first premolars and extraction of deciduous maxillary canine and deciduous mandibular canine bringing the impacted left mandibular canine in arch and finishing the case in Angles class I molar and canine relationship bilaterally. The other alternative plan was to extract the deciduous maxillary canine and deciduous mandibular canine along with impacted canine, followed by space closure to achieve ideal molar and canine relation with reshaping lower left mandibular premolar to canine. Another plan was to extract deciduous maxillary and deciduous mandibular canine and to bring the impacted transmigrated lower left canine back to occlusion. Out of these plans, the patient rejected the extraction plans of permanent teeth and wanted to go with extraction plan of deciduous teeth, which included getting impacted canine back to its original position.

Treatment progress

**Figure 5:**
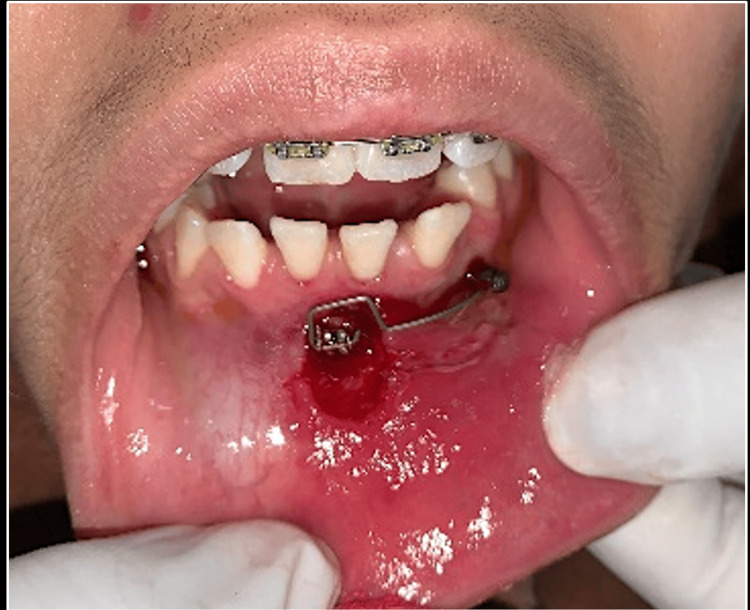
Uprighting spring made up of 0.017 x 0.025 TMA wire placed to the bracket the canine and TAD TMA: Titanium molybdenum alloy; TADs: temporary anchorage devices

The treatment started with the extraction of deciduous maxillary and mandibular canines. Orthodontic therapy was initiated with fixed appliance using 0.022 MBT (McLaughlin, Bennett, and Trevisi) bracket system, placed in the upper arch. Bonding was done and the initial 0.014NiTi (nickel titanium) wire was placed. In the next subsequent appointment, the impacted canine exposure was carried out using an open window technique. Once the exposure of transmigrated canine was achieved, a bracket was bonded to the canine. Inter-radicular temporary anchorage device (TAD) of size 1.5mm x 6mm was placed in the lower left quadrant at the canine and premolar regions. Since the impacted canine was close to the root surface of mandibular incisors, an uprighting spring (0.017 x 0.025 TMA (titanium molybdenum alloy)) was used from TAD to the exposed canine bracket for correcting the inclination and keep the canine away from the root of incisors (Figure 5).

It took around six months to correct the inclination of the canine. The uprighting spring was activated every month. While the inclination of impacted canine was being corrected, the maxillary arch progressed further from initial NiTi wires to stainless steel (SS) archwire as the treatment progressed (0.014 NiTi, 0.016 NiTi, 0.018 SS, 0.017 x 0.025 SS and 0.019 x 0.025 SS wire). Once the 0.018 SS archwire was placed, 0.012 NiTi archwire was given as a piggyback wire for repositioning of in-standing maxillary canine, which got aligned and got corrected as the treatment progressed.

**Figure 6:**
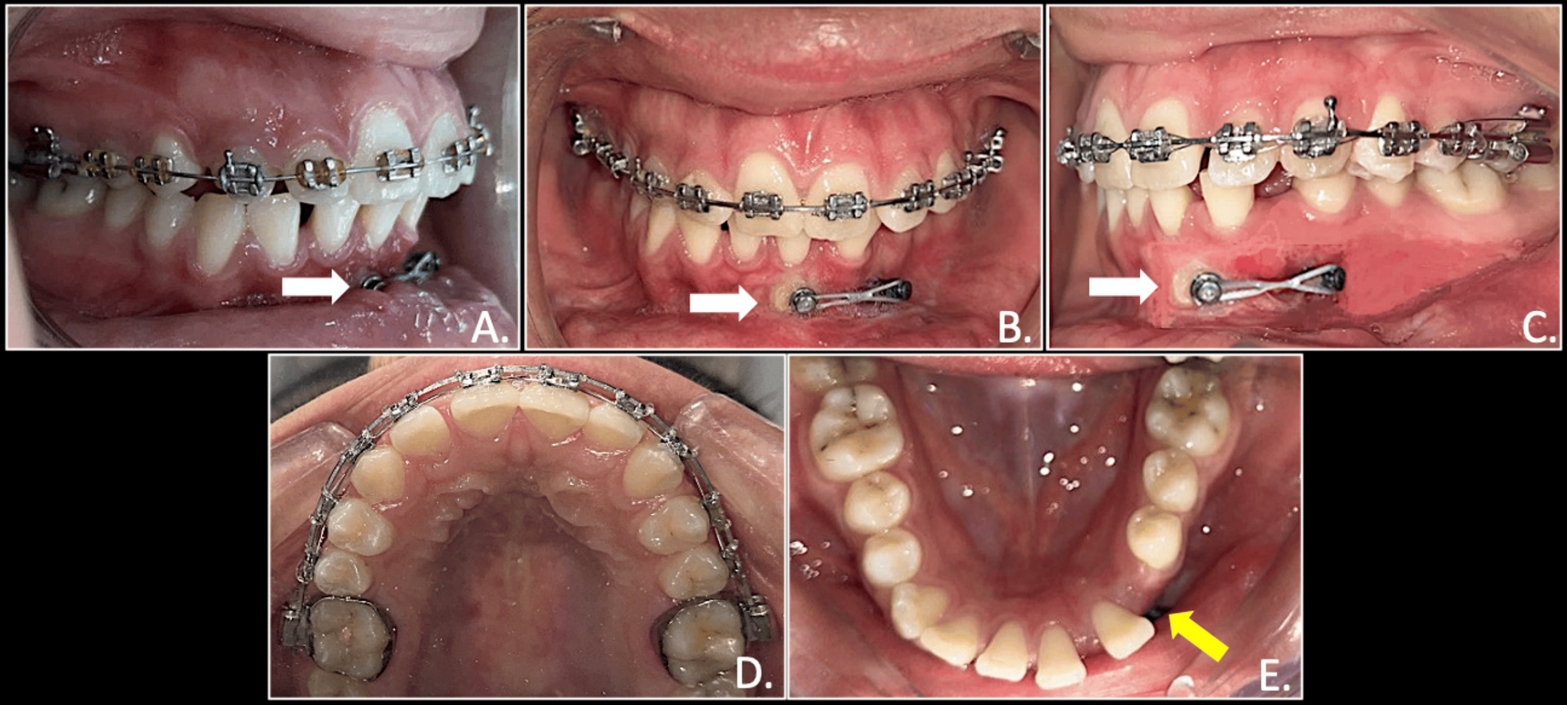
Elastic force traction for retraction of mandibular canine from TAD to round button on canine (white arrows): right occlusion (A), anterior in occlusion (B), left occlusion (C), maxillary arch (D), mandibular arch showing space available for permanent mandibular canine after extraction of deciduous canine (E) TADs: Temporary anchorage devices

Once the canine was upright, another TAD of size 1.5mm x 8mm was inserted between the first and second premolar. An elastomeric chain (e-chain) exerting force from TAD was attached to the canine for pulling in the desired direction (Figure 6).

**Figure 7:**
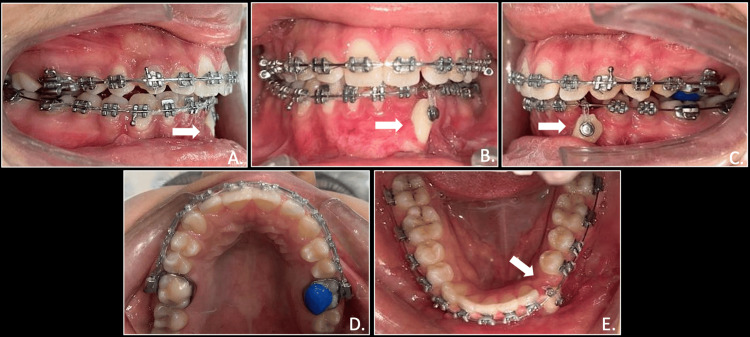
Elastic force traction of mandibular canine in vertical plane from rigid arch wire(white arrows): right occlusion (A), anterior in occlusion (B), left occlusion (C), maxillary arch (D), mandibular arch (E)

The e-chain was changed every four weeks and it took approximately three months for the canine to get repositioned in its original place. The lower arch was then bonded with 0.014 NiTi wire, followed by 0.016 NiTi, 0.018 SS, 0.017 x 0.025 SS, and 0.019 x 0.025 SS wire. Elastic force traction continued in vertical direction using modified biomechanics on rigid stainless steel wire. Once the transmigrated canine reached near the occlusal plane, a piggyback was given with 0.012 NiTi on 0.018 SS wire. This was followed by 0.014 NiTi for full mandibular arch and then wire sequence were followed from NiTi to SS wires including canine along with all lower teeth together. It was noticed that that the root positioning of the lower left canine brought into the mandibular arch was not appropriate. So, to correct it, root torquing was incorporated using Warren torquing spring, which was activated every month for two months on 0.019 x 0.025 SS wire till accurate root torque and positioning were achieved (Figure 7).

**Figure 8:**
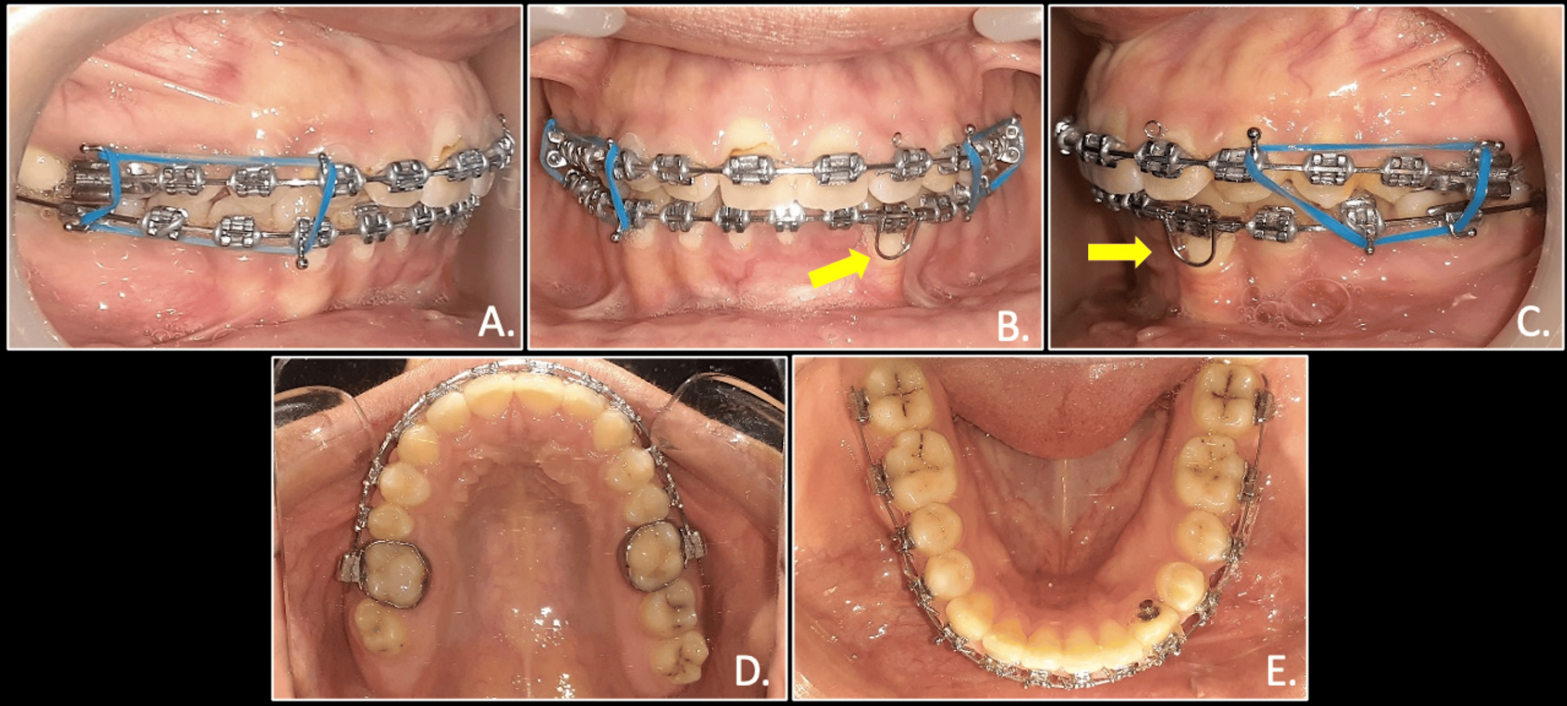
Finishing stage with settling elastics and Warren root torquing spring placed to provide lingual root torque for mandibular left canine: right occlusion (A), anterior in occlusion (B) (yellow arrows), left occlusion (C) (yellow arrows), maxillary arch (D), mandibular arch (E)

Further to correct the scissor bite with respect to the left side, arch coordination using AJ Wilcock stainless steel wires were done for maxillary and mandibular arches. Finally, settling elastics were given to achieve stable buccal occlusion and appropriate molar and canine relation (Figure 8).

Treatment results

**Figure 9:**
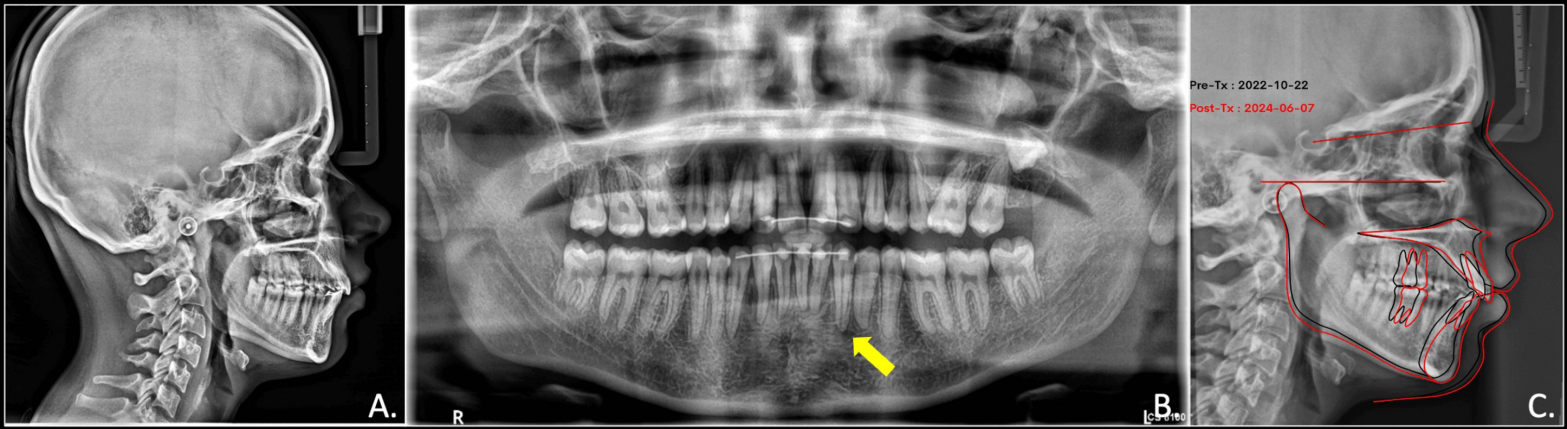
Post-treatment radiographs showing the final position of the mandibular canine and associated dental and bony components: lateral cephalogram (A), OPG showing root parallelism and adequate bony support around the canine (B) (yellow arrow). Pre and post-treatment cephalometric superimposition (C) OPG: orthopantomogram

The post-treatment assessment revealed favourable alignment, with class II molar and canine on the right and class 1 molar and canine on the left side with ideal overjet and overbite. The maxillary midline aligned with the facial midline, and a harmonious smile arc was achieved. The post-treatment radiographic evaluation revealed root parallelism of all the teeth with adequate bone support for the transmigrated canine. Post-treatment cephalogram analysis showed slightly proclined and forwardly placed maxillary as well as mandibular incisors. The pre-treatment and post-treatment cephalometric superimposition showed maintenance of dento-skeletal and soft tissue parameters (Figure 9).

**Figure 10:**
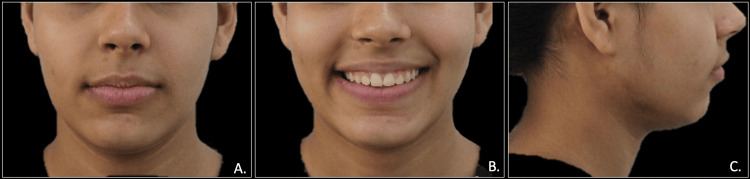
Post-treatment extraoral photographs: frontal view (A), frontal smiling view (B), and profile view (C)

**Figure 11:**
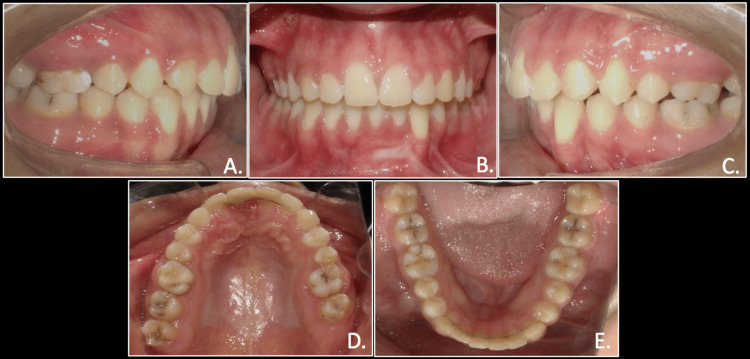
Post-treatment intraoral photographs: right occlusion (A), anterior in occlusion (B), left occlusion (C), maxillary arch (D), and mandibular arch (E)

The post-treatment assessment of the transmigrated canine revealed no further root resorption and no signs of mobility. The overall treatment lasted for one year and eight months, and the patient expressed satisfaction with the results. A lingual bondable permanent retainer was delivered in mandibular and maxillary arches (Figures 10, 11).

## Discussion

Mandibular canine transmigration is less frequent compared to maxillary canine impaction, occurring approximately 20 times less often. Mupparapu's classification categorizes canine transmigration considering their location and interaction with the surrounding teeth, identifying 45% as mesioangular, 20% as horizontal and situated beneath the tips of the mandibular incisors, and 1.5% emerging vertically along the midline [6]. While canine transmigration has been classified, clear guidelines are not available for its management. Wertz et al. in 1994 suggest that if the tip of a transmigrated canine extended past the adjacent incisor tooth, it may be challenging to utilise orthodontic treatment modalities to direct it into the proper alignment. In contrast, other authors propose extraction if the transmigrated canine is causing root resorption of the neighbouring teeth or is associated with a cyst [7].

In this case, the left mandibular canine was classified as type 1 by Mupparapu’s classification as it was impacted and positioned buccally and mesioangularly. Due to the retention of the primary deciduous canine and the partial resorption of its root, replacing the permanent canine with the primary canine was not feasible. Therefore, extracting the primary canine and guiding the transmigrated permanent canine into position was the most viable solution. Due to the unusual position of the mesioangularly impacted left canine, the treatment plan required complex three-dimensional orthodontic movements. To address this, modified biomechanics were employed, utilizing an uprighting spring (0.017 x 0.025 TMA) in conjunction with a miniscrew as an anchorage unit to correct the canine's inclination. Once the canine was upright, the mechanics were modified, and another miniscrew was placed between the lower left first and second premolars to retract the transmigrated canine.

After the retraction of canine, it was effectively integrated into the arch through elastic traction force, although it was challenging as the movement of the canine through the cortical bone required enhanced anchorage control, here provided by using TAD. To enhance the predictability of tooth outcomes movement and for appropriate biomechanical planning, three-dimensional CBCT imaging was done.

Due to the unpredictable onset of root resorption in impacted teeth, all such teeth should be considered at significant risk for external root resorption or harm to adjacent teeth. Regular radiographic assessments, such as orthopantomography, are essential for monitoring these risks [8,9]. Vuchkova and Farah reported in 2010 that over the past 50 years, 185 cases of transmigrated mandibular canines have been documented [10]. While several treatment options have been described, the most common approach involves extracting the transmigrated tooth. Following the extraction of the transmigrated tooth, the treatment plan typically involves either orthodontic correction of the malocclusion or no further treatment [4,11,12].

Given the uncertain outcome of repositioning transmigrated mandibular canines [13], Orthodontic-surgical repositioning of transmigrated mandibular canines has been sparsely documented in the literature [1,14,15]. Wertz in 1994 suggested that surgical repositioning of transmigrated mandibular canines should be considered when non-extraction orthodontic treatment is deemed appropriate [7]. A long-term follow-up study by Becker and Chaushu in 2005 indicates that surgical repositioning of transmigrated mandibular canines followed by extrusion with minimal orthodontic forces offers the most promising long-term outcome, as it effectively mitigates the risks of ankylosis and external root resorption [16]. A comprehensive review of the literature on transmigrate mandibular canine and modified orthodontic treatment used for successful disimpaction of canine is depicted in Table 1.

**Table 1:** Review of transmigrated mandibular canine cases

Reference	Transmigrated Mandibular Canine (Mupparapu Classification)	Side	Deviation of Transmigrated Canine from Midline	Mechanics Used
Pinto et al.(2020) [17]	Type 2	Right	Along the lower edge of mandible, under the roots of lower incisors	Extraction of transmigrated canine as associated with cyst.
Bhullar et al.(2017) [18]	Type 1	Left	At the midline, with crown close to roots of mandibular central incisors	Extraction of transmigrated canine.
	Type 1	Right	Along the lower edge of the mandible, under the roots of lower incisors	Extraction of transmigrated canine.
	Type 5	Right	At the midline, just apical to the roots of retained deciduous central incisors	Extraction of retained deciduous central incisors followed by surgical exposure and orthodontic traction of 43 in their place. Reshaping of canine as incisor was done at the end of the treatment.
Tarsariya et al.(2015) [19]	Type 6	Right	The canine impacted vertically beyond the body of the mandible on the contralateral side in the midramus region	No treatment
	Type 4	Right	Along the lower edge of the mandible, under the roots of the premolars incisors	
	Type 2	Right	Along the lower edge of the mandible, under the roots of lower incisors	
	Type 5	Right	Impacted vertically below the apices of incisors in midline	
Camilleri and Scerri (2003) [3]	Type 2	Right	Along the lower edge of the mandible, under the roots of lower incisors	No treatment
	Type 1	Right	At the midline, with the crown close to the roots of the mandibular central incisors	
	Type 2	Right	Along the lower edge of the mandible, under the roots of lower incisors	
	Type 1	Left	At the midline, with the crown close to the roots of the mandibular central incisors	

The primary objective of this case report was to evaluate the efficacy of a novel treatment approach for managing transmigrated mandibular canines. The treatment plan successfully achieved its goals, resulting in a satisfactory aesthetic outcome despite minimal gingival recession. The key factors contributing to the treatment's success were the judicious application of light continuous forces and the patient's unwavering motivation. Moreover, diagnosis using three-dimensional imaging played a pivotal role in preventing potential complications, particularly root resorption of adjacent teeth.

## Conclusions

The interdisciplinary management of transmigration by orthodontic-surgical synergy is a viable therapeutic modality. To mitigate potential iatrogenic damage to the teeth and supporting structures, application of light orthodontic forces and meticulous care are imperative. Dentists should expeditiously identify transmigrations and assess for orthodontic evaluation for comprehensive evaluation and treatment.
